# Polypseudorotaxanes of Pluronic® F127 with Combinations of α- and β-Cyclodextrins for Topical Formulation of Acyclovir

**DOI:** 10.3390/nano10040613

**Published:** 2020-03-27

**Authors:** Cristina Di Donato, Rosa Iacovino, Carla Isernia, Gaetano Malgieri, Angela Varela-Garcia, Angel Concheiro, Carmen Alvarez-Lorenzo

**Affiliations:** 1 Department of Environmental, Biological and Pharmaceutical Sciences and Technologies, University of Campania Luigi Vanvitelli, Via A. Vivaldi 43, 81100 Caserta, Italy; cristinadidonato7@gmail.com (C.D.D.); rosa.iacovino@unicampania.it (R.I.); carla.isernia@unicampania.it (C.I.); gaetano.malgieri@unicampania.it (G.M.); 2 Departament of Pharmacology, Pharmacy and Pharmaceutical Technology, I+DFarma (GI-1645), Facultad de Farmacia and Health Research Institute of Santiago de Compostela (IDIS), Universidade de Santiago de Compostela, 15782 Santiago de Compostela, Spain; angela.varela.garcia@rai.usc.es (A.V.-G.); angel.concheiro@usc.es (A.C.)

**Keywords:** Pluronic® F127, cyclodextrins mixture, acyclovir, polypseudorotaxanes, solubilization, controlled release

## Abstract

Acyclovir (ACV) is one of the most used antiviral drugs for the treatment of herpes simplex virus infections and other relevant mucosal infections caused by viruses. Nevertheless, the low water solubility of ACV limits both its bioavailability and antiviral performance. The combination of block copolymer micelles and cyclodextrins (CDs) may result in polypseudorotaxanes with tunable drug solubilizing and gelling properties. However, the simultaneous addition of various CDs has barely been investigated yet. The aim of this work was to design and characterize ternary combinations of Pluronic® F127 (PF127), αCD and βCD in terms of polypseudorotaxane formation, rheological behavior, and ACV solubilization ability and controlled release. The formation of polypseudorotaxanes between PF127 and the CDs was confirmed by FT-IR spectroscopy, X-ray diffraction, and NMR spectroscopy. The effects of αCD/βCD concentration range (0–7% *w*/*w*) on copolymer (6.5% *w*/*w*) gel features were evaluated at 20 and 37 °C by rheological studies, resulting in changes of the copolymer gelling properties. PF127 with αCD/βCD improved the solubilization of ACV, maintaining the biocompatibility (hen’s egg test on the chorio-allantoic membrane). In addition, the gels were able to sustain acyclovir delivery. The formulation prepared with similar proportions of αCD and βCD provided a slower and more constant release. The results obtained suggest that the combination of Pluronic with αCD/βCD mixtures can be a valuable approach to tune the rheological features and drug release profiles from these supramolecular gels.

## 1. Introduction

**Figure 1:**
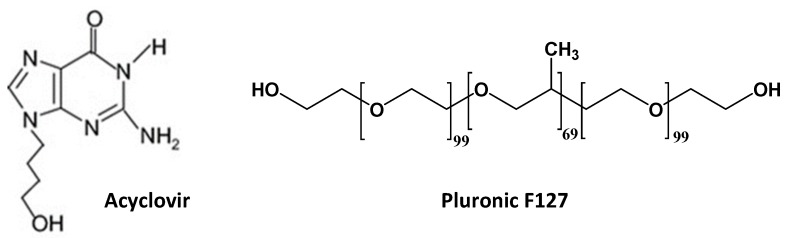
Chemical structure of acyclovir (ACV) and Pluronic F127 (PF127).

Acyclovir (ACV), 2-amino-1,9-dihydro-9-[(2-hydroxyethoxy)methyl]-6H-purin-6-one (Figure 1) is a purine nucleoside with an amphoteric behavior due to the presence of two ionizable groups that can be deprotonated or protonated as a function of the pH of the solution. Below pH 2.4, the basic group is charged positively, while the acidic group is charged negatively above pH 9.5. Thus, ACV neutral form predominates in the pH range of 3–8.5 [1]. ACV is a first line drug in the treatment of viral infections caused by herpes simplex viruses (HSV), Epstein–Barr virus (EBV), varicella zoster virus (VZV), and cytomegalovirus (CMV) [2,3]. After administration, ACV is phosphorylated, resulting in acyclovir triphosphate, which is able to interact with the viral DNA polymerase for inhibiting the replication. Relevantly, ACV is selective for viral enzymes and does not interfere with the activity of host cell DNA polymerase [4]. The drug is commonly marketed as tablets and topical cream, used primarily for labial herpes simplex, but it can also be administered intravenously and as ophthalmic ointment [5]. Although ACV is one of the most important antiviral drugs, its poor aqueous solubility [5] determines low oral bioavailability (15–20%). Moreover, its half-life is short (2–3 h). Therefore, new approaches in ACV formulation that improve its solubility and therapeutic efficacy are being investigated [6,7]. In this context, the use of polymeric micelles [8] or cyclodextrins [9,10,11] can be particularly suitable. Pluronic® F127 (Poloxamer 407, PF127), a poly(ethylene oxide) (PEO)− poly(propylene oxide) (PPO)− poly(ethylene oxide) (PEO) triblock, readily self-associates in aqueous medium forming micelle-like aggregates and even larger supramolecular structures leading to thermoreversible and mucoadhesive gels [12]. The copolymer shows unique sol-gel transition behavior in situ in response to temperature mediated by hydrophobic interactions among PPO blocks [13,14]. Furthermore, the presence of PEO, a mucoadhesive polymer, increases the surface interaction of the formulation with the tissue [15].

The addition of hydrophilic polymers to cyclodextrin (CD) solutions has been proposed as an opportunity to intensify the inclusion complex formation, improve the solubilizing capability, and modulate drug diffusion [16,17]. Nevertheless, some block copolymers can also penetrate in the CD cavity, forming necklace-like supramolecular structures, named polypseudorotaxanes, that can notably alter both the CD ability to solubilize drug and the sol-gel transition of the block copolymer [18,19]. In general, polypseudorotaxanes of block copolymers with αCD lead to semicrystalline 3D assemblies in which the PEO blocks are encapsulated in the αCD units and form reversible gels at much lower concentration than that required by the copolymer alone [20]. Inversely, block copolymers in the presence of βCD find self-assembly more difficult since the hydrophobic block is the one encapsulated by the βCD and thus the overall hydrophilicity of the copolymer increases, with less copolymer chains available for micellization [21,22].

Recently polypseudorotaxanes of Pluronic/Soluplus mixed micelles with αCD have been reported for another hydrophobic drug, such as natamycin; the combination of the two copolymers was revealed useful to tune the performance of the polypseudorotaxane in terms of drug diffusion and ocular permeability [23]. Combinations of one copolymer and a mixture of cyclodextrins to form hetero-polypseudorotaxanes have been much less investigated, but it has already been shown that adjustment of CDs ratio may regulate the solubility and the stability of the poly(pseudorotaxanes) [24,25,26]. To the best of our knowledge, drug formulation in systems combining PF127 (Figure 1) with αCD and βCD has not been reported yet. We hypothesized that mixing αCD and βCD may be an excellent tool to adjust Pluronic-based polypseudorotaxanes to specific demands as drug delivery systems, in terms of modifiable gelling conditions and gel consistency (opposed effects of αCD and βCD) and synergic drug solubilizing capability (αCD and βCD may form complexes with different molecules and polymer moieties), which in turn may determine drug release patterns. Thus, the aim of this work was to design and characterize ternary combinations of PF127, αCD, and βCD covering a wide range of weight ratios in terms of polypseudorotaxane formation, rheological behavior, and ACV solubilization ability and controlled release.

## 2. Materials and Methods

### 2.1. Materials

All reagents and solvents were of analytical grade and used without prior purification. MilliQ® water was used throughout the experiments. Pluronic® F127 (PF127, EO_100_–PO_69_–EO_100_, 12,600 Da) was purchased from Sigma–Aldrich (St. Louis, MO, USA), αCD and βCD from Wacker (Burghausen, Germany), and ACV from Roig Pharma (Barcelona, Spain). The sodium citrate/citric acid buffer solution was prepared as reported in the European Pharmacopoeia [27], and its pH was fixed at 4.9 (pH-meter CRISON, model GLP22, Barcelona, Spain).

### 2.2. Gel Preparation

PF127 solution at 13% *w*/*w* was prepared according to the “cold method” [28]. Specific amounts of copolymer were added to citrate buffer. The solution was kept under stirring in an iced bath for 24–36 h until it became clear, and then stored at 4 °C. Gels containing ACV were similarly prepared by adding 4.5 g ACV to 40.5 g of PF127 (13% *w*/*w*) solution and kept under stirring for 24–36 h. The αCD/βCD dispersions (0–14% *w*/*w* total concentration) were prepared adding specific amounts of each CD to citrate buffer and kept under heating (50 °C) for several hours. Then, 5 mL of PF127 (13% *w*/*w*) solution was mixed with 5 mL of αCD/βCD solution to obtain systems covering a wide range of weight ratios (Table 1). They were immediately transferred to 4 °C for 24 h and then stored at room temperature for at least 48 h before measurements.

### 2.3. X-Ray Diffraction and FT-IR Spectroscopy

XRD diffraction patterns of powders obtained from PF127-CDs dispersions dried at 50 °C for three days were recorded at room temperature using a powder diffractometer fitted with Philips PW1710 control unit, vertical Philips PW1820/00 goniometer and FR590 Enraf Nonius generator. The instrument was equipped with a graphite diffracted beam monochromator and copper radiation source (*λ*(Kα1) = 1.5406Å), operating at 40 kV and 30 mA. The X-ray powder diffraction patterns (XPRD) were collected by measuring the scintillation response to Cu Kα radiation versus the 2θ value over a 2θ range of 3–30°, with a step size of 0.02° and counting time of 2 s per step.

FT-IR spectra were recorded using KBr disks in a FTIR Varian 670-IR. The scanning range was 4000–500 cm^−1^ with a resolution of 4 cm^−1^.

### 2.4. NMR Studies

Formulations reported in Table 1 were dried at 40 °C and the obtained powders were dissolved in D_2_O (Sigma-Aldrich, St. Louis, MO, USA). All spectra were recorded on a Bruker Avance III HD 600  MHz equipped with cryoprobe. The ^1^D spectra were acquired with 16 K and zero-filled to 32 K data points for each preparation. ^1^H chemical shifts were recorded at 300 K, at pH = 4.9, and referenced to external TMS (*δ* = 0 ppm). Although two-dimensional phase-sensitive NOESY (Nuclear Overhauser Effect SpectrscopY) and ROESY (Rotating frame Overhauser Effect SpectroscopY) spectra were collected using the States and Haberkorn method [29] with mixing times of 100 and 150 ms, respectively, no significant Nuclear Overhauser Enhancements were detected. Water suppression, when necessary, was achieved using the DPFGSE (Double Pulsed Field Gradient Spin Echo) sequence [30]. The hydrodynamic properties of all samples were estimated using the translational diffusion coefficient (*Dt*) measured by pulsed-field gradient spin-echo DOSY(Diffusion Ordered SpectroscopY) experiments [31], with time interval (*Δ*) set to 100 ms and gradient pulse (*δ*) to 4 ms. A total of 32 gradient increments, linearly varied from 2% to 95%, were collected with 32 scans for each increment. Typically, 64 transients of 4 K data points were collected for each of the 256 increments; the data were zero filled to 1 K in ω1. Squared shifted sine-bell functions were applied in both dimensions prior to Fourier transformation and baseline correction. The data were processed and analyzed using the TopSpin 3.5 (Bruker, Billerica, MA, USA) and CARA software (version 1.7.1, downloaded from cara.nmr.ch) [32].

### 2.5. Rheological Studies

The rheological behavior of the PF127-CD dispersions was evaluated using a Rheolyst AR-1000 N rheometer (TA Instruments, New Castle, UK), equipped with an AR2500 data analyzer and fitted with a Peltier plate. The storage (*G’*) and loss (*G”*) moduli were recorded at 20 and 37 °C using a cone-plate geometry (diameter 6 cm, angle 2°) applying a frequency sweep in the 0.10–50 rad/s range. Furthermore, the moduli were recorded at 5 rad/s in a temperature ramp of 2 °C/min from 20 to 37 °C.

### 2.6. Phase Solubility Studies

ACV was added in excess (50 mg) to vials containing 8 mL of PF127 (2–13%) or αCD/βCD (0–7%) solutions prepared in citrate buffer at pH 4.9. The vials were then sealed and thermostatically shaken at 25 °C for the PF127 solutions and at 40 °C for CDs solutions for five days. This amount of time was sufficient to reach equilibrium [33,34]. Subsequently, aliquots were filtered using a syringe through 0.22 μm Millipore® cellulose acetate membrane filters (Teknokroma, Sant Cugat del Vallès, Spain), diluted in water/ethanol 50:50 vol/vol, and analyzed by UV spectrophotometry at 252 nm. All experiments were performed in triplicate.

### 2.7. HET-CAM Assay

The HET-CAM (Hen’s Egg Test Chorio Allantoic Membrane) test was performed using fertilized hen’s eggs (50–60 g; Coren, Santa Cruz de Arrabaldo, Spain) incubated at 37 °C and 60% relative humidity for 8 days, as described previously [35]. Then, a circular cut of 1 cm in diameter was made on the wider edge of each egg (air chamber) and the inner membrane was moistened with NaCl 0.9% solution for 30 min. Then, the NaCl 0.9% solution and the membrane were removed to expose the chorioallantoic membrane (CAM). Volumes of 300 μL of PF127 6.5% + ACV 5% and PF127 6.5% – αCD/βCD + ACV 5% dispersions (compositions as in Table 1) were placed on the CAM of various eggs. Also, NaCl 0.9% and NaOH 0.1 N solutions were used as negative and positive controls, respectively. The vessels of CAM were monitored for 5 min, under white light, to record the time of appearance of hemorrhage (*T*h), vascular lysis (*T*l), or coagulation (*T*c). The irritation score (*IS*) was calculated, as previously reported [35], as follows

*IS* = [(301 − *T*h) × 5/300] + [(301 − *T*l) × 7/300] + [(301 − *T*c) × 9/300]

### 2.8. ACV Release Tests

Permeation studies were carried out in triplicate, with ACV 2% solubilized in buffer, PF127 6.5%, PF127 6.5% + βCD 7%, PF127 6.5% + αCD 3%/βCD 4%, PF127 6.5% + αCD 5%/βCD 2%, and PF127 6.5% + αCD 7% formulations. The receptor compartment of the Franz cell was filled with citrate buffer pH 4.9 (6 mL), which was kept under magnetic stirring at 200 rpm and 37 ± 1 °C. A cellulose nitrate membrane of 0.45 μm with a diameter of 25 mm was placed between the donor and the receptor compartment (area available for permeation: 0.785 cm^2^). Aliquots (1 mL) of each solution were poured in the donor compartments. At given time intervals for six hours, samples (1 mL) of receptor medium were taken and immediately replaced with an equal volume of fresh citrate buffer. The absorbance of the samples was measured at 252 nm (UV/VIS spectrophotometer Agilent 8453, Waldbronn, Germany) and ACV concentration was calculated after suitable dilution in ethanol:water 50/50 *v*/*v* medium. The permeability coefficient, *Peff*, was estimated from the slope of the amount of ACV in the receptor compartment per unit area versus the time, for the first four hours of the experiment. The diffusion coefficient, *D*, was estimated from the slope of the plots of the amounts of ACV in the receptor compartment versus the square root of time as follows [36]:

M/A = 2·C_0_·(D/π)^0.5^·t^0.5^

where *M* is the amount of ACV accumulated in the receptor compartment, *A* is the diffusion area, and *C*_0_ is the concentration of ACV in the formulation.

### 2.9. Statistical Analysis

Differences in ACV solubility, *Peff* and *D* among the formulations were analyzed using ANOVA followed multiple range test (Statgraphics Centurion 18, StatPoint Technologies Inc., Warrenton, VA, USA).

## 3. Results and Discussion

### 3.1. Polypseudorotaxanes Formation and Characterization in Solid State

As soon as the Pluronic and αCD/βCD dispersions were mixed, the systems became progressively turbid dispersions or white gels. It should be noted that the starting αCD and βCD dispersions at 14% were not solutions but fine suspensions, and therefore the mixtures were prepared while keeping the suspensions heated at 50 °C under stirring for a homogeneous sampling of the aliquouts. Heating favored CD solubility and the inclusion of the block copolymer into the CD cavity [22]. Systems containing the highest αCD concentrations tested (6% or 7%) transformed into gels either at 4 °C or at room temperature in a few hours, but two phases were macroscopically observed after 48 h storage: a clear low viscosity phase on the top of a white gel phase. In contrast, as the proportion of βCD increased, the systems did not show separation in two macroscopic phases but the white viscous gel became less viscous. The visualization of two phases in the PF127/αCD 7% system suggested that the polypseudorotaxanes were somehow collapsed and could not form a complete 3D supramolecular gel filling the whole volume of the dispersion. This phase separation was not observed in previous reports on PF127/αCD polypseudorotaxanes prepared in pure water [37,38,39,40] and may be caused by an enhanced aggregation of the αCDs in the buffer pH 4.9 used to prepare the formulations. This acid pH adversely affects the solubility of the αCDs but is preferable for topical skin formulations. It should be also noted that in the PF127 6.5%–αCD 7% system the EO:αCD molar ratio was approx. 14:1, while in the PF127 6.5%–βCD 7% system the PO:βCD molar ratio was approx. 5.6:1. Both molar ratios were well above the 2:1 stoichiometry reported for EO:αCD and PO:βCD complex formation [22,41], which means that there are CD-free regions in the copolymer chains. Therefore, the designed supramolecular gels may be formed by a mixture of polymeric nanomicelles and aggregates of nanosized polypseudorotaxanes that assemble forming interconnected macrostructures [22,41,42].

**Figure 2:**
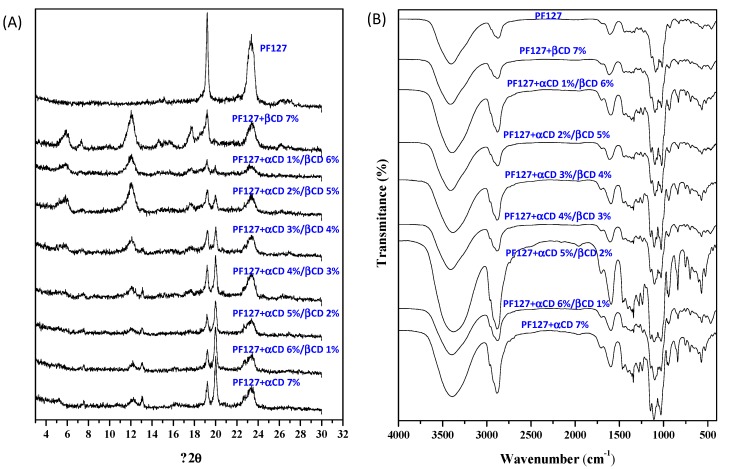
The XRD patterns (**A**) and FT-IR spectra (**B**) of dried powders obtained from PF127 solely dispersions and PF127 dispersions with αCD/βCD at different concentration ratios.

Samples of the prepared formulations were dried and first characterized by means of X-ray diffraction (XRD) and FT-IR spectroscopy. The inclusion complex formation between PEO and PPO blocks of the Pluronic with αCD and βCD, respectively, was confirmed with wide-angle X-ray diffraction studies. The characteristic peaks of PF127 (Figure 2A) appeared at diffraction angles of 19.19 and 23.31° 2 θ [37]. In presence of high concentrations of αCD (i.e., 7% αCD and αCD 6%/βCD 1%) the formulation showed well defined peaks at 12.9° and 19.9°, which are characteristic of a channel type crystalline structure with αCD units organized head-to-head and tail-to-tail, typical for polypseudorotaxanes [38,39,42]. The intensity of these peaks decreased as the βCD relative concentration increased. PF127 with αCD 2%/βCD 5% and αCD 1%/βCD 6% showed attenuated crystallinity, and the PF127-βCD 7% spectrum corresponded to the amorphous state (Figure 2A). Therefore, the channel type crystalline structure correlated to the well-known ability of αCD to form selective and stable inclusion complexes only with EO units of Pluronic®, while the presence of amorphous aggregates at higher concentrations of βCD confirmed that this cyclodextrin better fits with the PO units [26].

FT-IR spectroscopy provided further information on the type of the interaction between PF127 and CDs [36]. The FT-IR spectrum of PF127 (Figure 2B) showed characteristic absorption bands in the 2888–3439 cm^−1^ region due to the stretching of the –OH groups. The absorption between 1467 and 1716 cm^−1^ was assigned to the bending vibration of –CH bonds, while the bands at 1112, 1147, 1360 cm^−1^ were due to the C–O bonds. The peaks at 1716, 1147, and 1360 cm^−1^ appeared with a weak intensity in the spectra of PF127 with high concentrations of βCD. These bands are likely correlated to the PO units, confirming that βCD cavity has higher affinity for this block. Furthermore, the peaks in the region between 2888 and 3439 cm^−1^ became broader as αCD concentration increased (Figure 2B), which could be due to the stretching bands for the EO units included in the αCD cavity [28]. Therefore, the FT-IR spectroscopy results agree well with those obtained from XRD and indicate the formation of two different species of polypseudorotaxanes that coexist when αCD and βCD are simultaneously present.

### 3.2. NMR Studies

**Figure 3:**
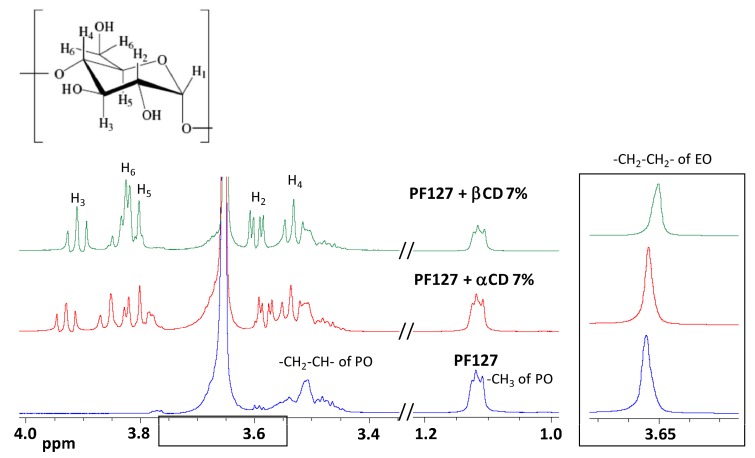
^1^H-NMR spectra of PF127 6.5% solely and with αCD 7% or βCD 7%. The insert shows the expanded region assigned to –CH_2_–CH_2_– of EO. The scheme of CD monomer stereo-configuration is also reported.

NMR studies were carried out for PF127 6.5% dispersion in the presence and the absence of αCD or βCD 7% (Figure 3). The dispersions were prepared in citrate buffer (Table 1), dried and redispersed in D_2_O. We have previously observed that the drying step does not significantly modify the NMR results in this kind of systems [40]. The use of D_2_O instead of H_2_O:D_2_O mixtures may lead to faster polypseudorotaxane formation kinetics, but the final architecture of the polypseudorotaxanes is expected to be almost the same [43]. The spectrum of PF127 had three main groups of peaks at 3.654 ppm, 3.514 ppm, and 1.118 ppm assigned to –CH_2_–CH_2_– of EO and to −CH_2_–CH– and –CH_3_ of the PO portion, respectively (Figure 3). The translational diffusion coefficient (*Dt*) was measured and resulted to be equal to 1.09 × 10^−10^ m^2^ s^−1^. *Dt* offers information about the molecular organization as it depends on the molecule size that can be calculated from the Stokes–Einstein equation. This value was slightly higher than that previously reported for related block copolymers [40]. In the presence of both cyclodextrins, the chemical environment surrounding these groups changed, confirming that the inclusion phenomenon took place.

In particular, PF127 with αCD 7% showed a chemical shift of the PEO signal from 3.654 to 3.653 while the chemical shift and the hyperfine structure of PPO signals remained unchanged, confirming the preferential interaction of PEO blocks with αCD [41]. Accordingly, the DOSY spectrum recorded for PF127 with αCD 7% confirmed the interaction of the αCD with the copolymer since the measured αCD *Dt* was 4.45 × 10^−10^ m^2^ s^−1^, which is consistent with an inclusion complex formation [39,44]. Differently, addition of βCD 7% caused shifts in the three groups of signals of PF127 to 3.650, 3.506, and 1.115 ppm. The DOSY experiment spectra also confirmed host-guest interactions; the measured *Dt* from the βCD signals was 4.26 × 10^−10^ m^2^ sec^−1^ [38]. These data indicate that βCD may form inclusion complexes with both blocks of PF127 [22].

**Figure 4:**
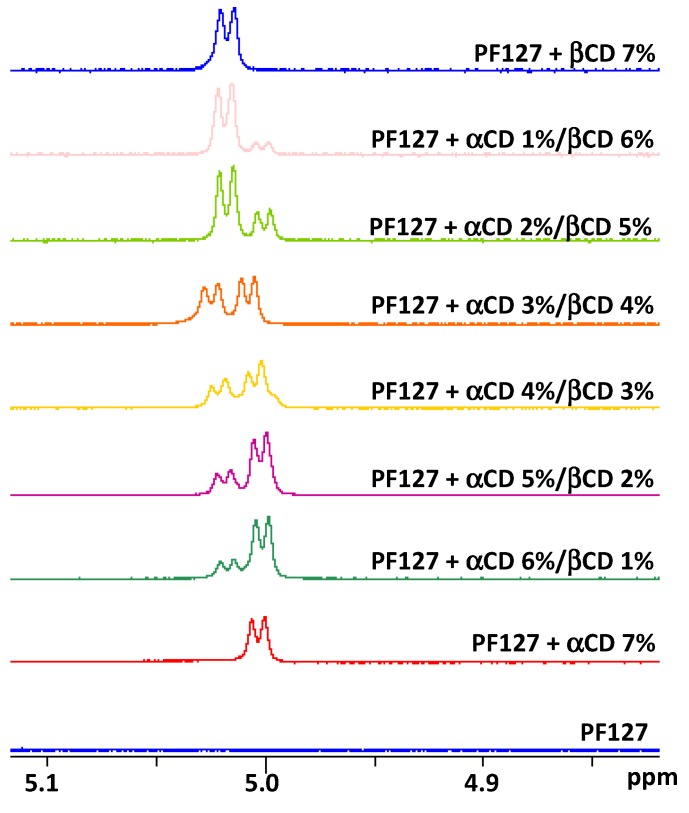
^1^H NMR spectra showing the CD H1 signals.

Ternary PF127 + αCD/βCD systems spectra are shown in Figure 4. The α/β ratio in each system was evident in the intensities of the CD H1 signals at ca. 5 ppm: the signal at higher field belonged to αCD, while the one at lower field to βCD. These signals did not show significant changes in the chemical shift in the different formulations.

In the presence of increasing concentrations in βCD, the PF127 PPO signals progressively shifted towards the values of the complex formed with βCD 7%, indicating that an increasing proportion of PPO groups became complexed as the βCD concentration augmented. The PEO signal, in presence of different α/β ratios, showed chemical shifts with intermediate values between the chemical shift recorded when in presence of αCD 7% and the one recorded when in presence of βCD 7% (Figure 3). In the different formulations, both CDs showed similar diffusion coefficients thus demonstrating that both were interacting with PF127. Altogether, these data indicate that, while the PPO portion is threaded into βCDs, the PEO portion of the polymer may interact simultaneously with both CDs depending on the α/β ratio, which confirms the formation of hetero-polypseudorotaxanes.

### 3.3. Rheological Properties

**Figure 5:**
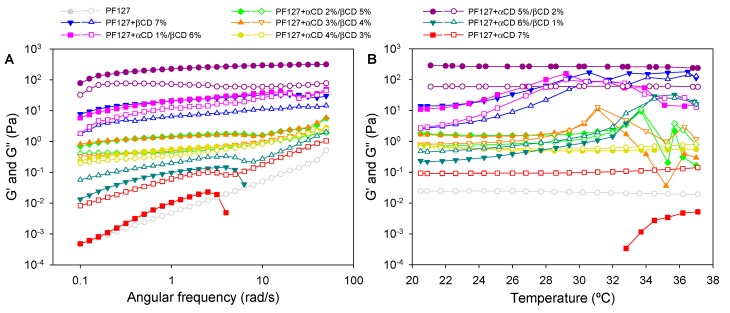
Storage (*G’*, full symbols) and loss (*G”*, open symbols) moduli of the PF127-αCD/βCD formulations recorded (**A**) at 20 °C as a function of the angular frequency, and (**B**) at 5 rad/s as a function of the temperature.

Effects of the hetero-polypseudorotaxane formation on the viscoelastic properties of the PF127 systems were then investigated (Figure 5). In the absence of CDs, PF127 6.5% solely dispersion behaved as a viscous system showing low *G”* values and negligible *G’*. PF127 dispersions require higher copolymer concentration to undergo sol-to-gel transition at physiological temperature [14,45,46]. Thus, polypseudorotaxane formation may enhance the consistency and the permanence on the application site of formulations comprising a relatively low concentration of block copolymer, which may be a safer strategy. Furthermore, polypseudorotaxane gels erode more slowly under physiological conditions than copolymer-solely gels [47].

In the presence of αCD 7%, *G”* increased by one order of magnitude at 20 °C and *G’* become evident when the angular frequency increased. However, a sudden decrease in the viscoelasticity occurred at 5 rad/s, which meant that the polypseudorotaxane gel became disassembled. PF127 + αCD 6%/βCD 1% system showed a slight increase in *G’* and *G”*. In contrast, PF127 + αCD 5%/βCD 2% formulation exhibited a remarkable increase in viscoelasticity, performing as a well-structured gel with *G’* values above *G”*, showing no dependence on angular frequency (Figure 5A) or temperature (Figure 5B). As mentioned, above this formulation was the one with the highest content in αCD that did not undergo macroscopic phase separation. Therefore, macroscopically homogeneous and stable gels were obtained. Further decrease in αCD and increase in βCD (αCD/βCD 4/3, 3/4 and 2/5%) led to intermediate viscoelastic behavior at 20 °C, performing as weak gels with *G’* values still above *G”* values. Polypseudorotaxane gels prepared with the highest content in βCD (αCD/βCD 1/6 and 0/7%) showed a small increase in *G’* and *G”* values, which could be related to the aggregation of the polypseudorotaxanes as platelets, as previously described for Pluronic F68 and βCD dispersions in water prepared following a similar protocol [22].

Interestingly, during heating from 20 to 37 °C formulations prepared with a βCD concentration of 4% or above suffered a remarkable fall in *G’* and *G”* at temperature close to 30 °C (Figure 5B). The formulation lost the gel appearance and appeared as a fluid system with small aggregates (insoluble clusters) [22]. It should be noted that in these formulations βCD units may have encapsulated most PO units of the copolymer, and therefore, temperature-induced self-aggregation of the copolymer is disrupted. Differently, formulations with a predominance of αCD (e.g., αCD/βCD 6/1), in which PO units remained free, exhibited a small increase in *G’* and *G”* values with temperature. Overall, the hetero-polypseudorotaxane formulations in which *G’* and *G”* values remained nearly constant in the temperature range evaluated were those prepared with αCD/βCD 5/2 and 4/3%.

### 3.4. Phase Solubility Studies

The intrinsic solubility of ACV in water was 1.2 mg/mL as reported in literature [10], while the solubility in the buffer pH 4.9 resulted to be 27.4 mg/mL (i.e., 0.12 M). The solubility values of ACV in PF127 dispersions and in solutions of αCD/βCD at different weight ratios are summarized in Table 2. Only the formulation prepared combining αCD 4%/βCD 3% increased the apparent solubility of ACV (36.0 mg/mL, 0.16 M). This means that ACV was not prone to be encapsulated in PF127 polymeric micelles in agreement with studies carried out with other amphiphilic copolymers [48]. Inclusion complex formation of ACV with CDs, although feasible, has been reported to cause minor enhancements in apparent solubility [49].

### 3.5. HET-CAM Test

HET-CAM (Hen’s Egg Test Chorio-Allantoic-Membrane) assay is an useful approach to evaluate the cytotoxicity and the irritation of chemicals, considering that the test mimics vascular changes in the chorioallantoic membrane, an analog for ocular conjuntiva [50,51]. Furthermore, this approach can be useful to evaluate primary cutaneous irritation also for topical formulations, providing indices of biocompatibility for a given material [52]. Control studies using 0.9% NaCl as a negative control led to IS values of 0.0, while with respect to the 0.1 N NaOH as positive control, the IS value was 21. Values below 0.9 indicate non-irritant behavior, while above 10 correspond to highly irritant substances. All formulations performed as the negative control, which means that they do not cause hemorrhage, vascular lysis, or coagulation, and thus may be tolerated by ocular tissues and skin.

### 3.6. ACV Release Tests

**Figure 6:**
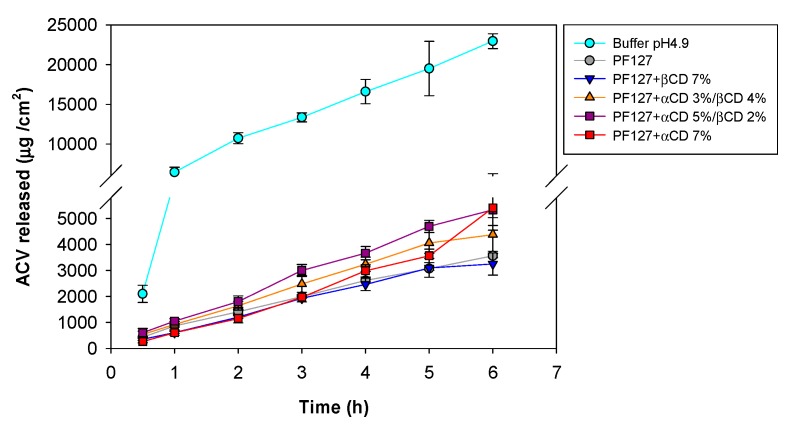
Acyclovir diffusion profiles from citrate buffer, PF127 6.5% dispersion, and PF127 6.5% polypseudorotaxane formulations with αCD/βCD at various ratios.

Diffusion of ACV from the formulations was investigated for a fix drug concentration of 20 mg/mL in order to ensure its complete solubilization. The experiments were recorded in Franz vertical cells. Compared to the buffer, all formulations showed more sustained release (Figure 6). The permeability coefficient, *Peff*, was estimated from the slope of the amount of ACV released per unit area versus the time for the first four hours of the experiment, while the diffusion coefficient, *D*, was estimated from the slope of the plots of the amount of ACV released per unit area versus the square root of time for the whole test period (goodness of the fitting, *r* > 0.98) (Table 3).

The gel formulations showed significantly lower values of *Peff* and *D* compared to the free ACV dispersed in the buffer solution (F_5,12_ = 43.25; *p* < 0.0001). A detailed comparison among formulations also revealed statistically significant differences (F_4,10_ = 14.89; *p* < 0.0003). Specifically, *D* values recorded for PF127 6.5% dispersion and PF127 + βCD 7% polypseudorotaxane formulation were significantly lower than those recorded for PF127 + αCD 5%/βCD 2% and PF127 + αCD 7% polypseudorotaxane formulations (*p* < 0.001). The PF127 + αCD 3%/βCD 4% formulation showed an intermediate behavior. This means that ACV moves faster in the gels in which αCD plays the main role in the polypseudorotaxane formation; that is to say, the gels in which there are more three-dimensional connections among the ends of the Pluronic chains. This network may have large pores between Pluronic chains as they are forced to be extended due to the threading of the αCD units along the PEO blocks to subsequently form inter-polypseudorotaxane tie-junctions. Differently, in the PF127 6.5% dispersion and PF127 + βCD 7% polypseudorotaxane formulation the dominant species should be individualized micelles and a mixture of micelles and unimers threaded by βCD, respectively. A high concentration of movable bulky species seems to hinder more efficiently the movement of ACV in spite of what was seen in the macroviscosity of the formulations; namely, *G’* and *G”* values of PF127 + αCD 5%/βCD 2% formulation were larger than those recorded for other formulations but the *D* values of ACV were larger too. This behavior agrees with previous reports highlighting the fact that macroviscosity does not always correlates with the microviscosity (i.e., resistance of the medium to the diffusion of small molecules) [53]. Indeed, it has been shown that the cross-linking of a polymer may lead to a rapid rise in macroviscosty while the movement of small molecules through the network is not altered since they move through the so called “interconnected water pools” among the chains [54].

## 4. Conclusions

Inclusion complex formation between PF127 and αCD/βCD was confirmed by FT-IR spectroscopy, X-Ray diffraction and NMR spectroscopy, which revealed the feasibility of hetero-polypseudorotaxanes formation. The relative proportion of αCD/βCD used to prepare the polyspeudorotaxanes had a strong impact on the rheological properties of the formulations. Maximum loss and storage moduli and also gel stability were obtained when an adequate balance between the αCD-induced polypseudorotaxane assembly and the βCD-induced polypseudorotaxane solubilization was achieved. This finding was observed for PF127 at 6.5% when mixed with 5% αCD and 2% βCD. Interestingly, although minor improvements in ACV solubility were observed, the different inner architecture of the hetero-polypseudorotaxanes gels determined their capability to regulate drug release rate. The diffusion coefficients did not obey an inverse correlation with the macroviscosity, but they were dependent on the components arrangements at the micro-scale. The faster ACV diffusion recorded for gels in which the αCD/βCD ratio was high suggest that there are larger water interconnect pores in the αCD-driven polypseudorotaxane 3D networks than in the less interconnected βCD-based polypseudorotaxane gels. Since the Pluronic + αCD/βCD formulations have successfully passed the preliminary biocompatibility tests, hetero-polypseudorotaxane formation can be positioned as a suitable tool to regulate rheological properties and drug release from topical formulations.

## Figures and Tables

**Table 1:** Composition of gels prepared with PF127 and αCD/βCD mixtures with and without ACV.

Pluronic® F127 (%, *w*/*w*)	αCD (%, *w*/*w*)	βCD (%, *w*/*w*)	ACV (%, *w*/*w*)
6.5	0	0	0/5
6.5	7	0	0/5
6.5	6	1	0/5
6.5	5	2	0/5
6.5	4	3	0/5
6.5	3	4	0/5
6.5	2	5	0/5
6.5	1	6	0/5
6.5	0	7	0/5

**Table 2:** Solubility of ACV in presence of buffer, PF127 micelles and αCD/βCD solutions at different weight ratios expressed with their standard deviations (SDs). Mean values and, in parenthesis, standard deviations (n = 3).

Medium	ACV Apparent Solubility (mg/mL)
Buffer pH 4.9	27.4 (0.1)
PF127 2%	18.9 (0.5)
PF127 3%	19.7 (0.3)
PF127 4%	18.8 (1.4)
PF127 6.5%	16.7 (0.2)
PF127 8%	14.8 (0.2)
PF127 13%	12.6 (1.3)
αCD 7%	22.5 (2.8)
αCD 6%/βCD 1%	26.4 (1.1)
αCD 5%/βCD 2%	24.7 (0.1)
αCD 4%/βCD 3%	36.0 (0.1)
αCD 3%/βCD 4%	26.7 (0.1)
αCD 2%/βCD 5%	26.3 (0.1)
αCD 1%/βCD 6%	25.9 (0.1)
βCD 7%	21.7 (3.6)

**Table 3:** Permeability coefficient, *Peff*, and diffusion coefficient, *D*, of ACV. Mean values and, in parenthesis, standard deviations (*n* = 3).

Formulation	*Peff* (cm/s)	*D* (cm^2^/s)
Buffer pH 4.9	54.3 × 10^−6^	70.9 × 10^−6^ (17.8 × 10^−6^)
PF127 6.5% dispersion	8.4 × 10^−6^	1.76 × 10^−6^ (0.09 × 10^−6^)
PF127 + βCD 7%	8.6 × 10^−6^	1.77 × 10^−6^ (0.43 × 10^−6^)
PF127 + αCD 3%/βCD 4%	10.7 × 10^−6^	2.95 × 10^−6^ (0.63 × 10^−6^)
PF127 + αCD 5%/βCD 2%	12.5 × 10^−6^	4.24 × 10^−6^ (0.59 × 10^−6^)
PF127 + αCD 7%	10.6 × 10^−6^	5.38 × 10^−6^ (0.13 × 10^−6^)
